# Gene expression in the liver of female, but not male mice treated with rapamycin resembles changes observed under dietary restriction

**DOI:** 10.1186/s40064-015-0909-7

**Published:** 2015-04-11

**Authors:** Zhen Yu, Bharath Sunchu, Wilson C Fok, Nahla Alshaikh, Viviana I Pérez

**Affiliations:** Linus Pauling Institute, Oregon State University, 307 Linus Pauling Science Center, Corvallis, OR 97331 USA; Department of Biochemistry and Biophysics, Oregon State University, Corvallis, OR 97331 USA; Department of Medicine, Division of Hematology, Washington University in St. Louis, St. Louis, MO 63110 USA

**Keywords:** Rapamycin, Dietary restriction, Gene expression, Female mice, Longevity pathways

## Abstract

It is well known that in mice the extension in lifespan by rapamycin is sexually dimorphic, in that it has a larger effect in females than males. In a previous study we showed that in male C57BL6 mice, rapamycin had less profound effects in both gene expression and liver metabolites when compared to dietary restriction (DR), but no data was available in females. Because recent studies showed that rapamycin increases longevity in a dose dependent manner and at every dose tested the effect remains larger in females than in males, we hypothesized that rapamycin should have a stronger effect on gene expression in females, and this effect could be dose dependent. To test this hypothesis, we measured the changes in liver gene expression induced by rapamycin (14 ppm) with a focus on several genes involved in pathways known to play a role in aging and that are altered by DR. To investigate whether any effects are dose dependent, we also analyzed females treated with two additional doses of rapamycin (22 and 42 ppm). We observed striking differences between male and female in gene expression at 14 ppm, where females have a larger response to rapamycin than males, and the effects of rapamycin in females resemble what we observed under DR. However, these effects were generally not dose dependent. These data support the notion that female mice respond better to rapamycin, and at least with the set of genes studied here, the effect of rapamycin in females resemble the effect of DR.

**Electronic supplementary material:**

The online version of this article (doi:10.1186/s40064-015-0909-7) contains supplementary material, which is available to authorized users.

## Introduction

Several studies published in the last few years indicate that rapamycin extends lifespan in different species, including the study by the NIA Intervention Testing Program (ITP), which reported that rapamycin increases lifespan in mice when the treatment started at either 9 or 20 months (Harrison et al. 2009), and the result is dose-dependent (Miller et al. 2014). Rapamycin is a clinically approved drug known to inhibit the mammalian Target of Rapamycin (mTOR), a serine/threonine kinase that regulates the response of eukaryotic cells to nutrients, growth factors, and cellular energy status. Interestingly, it has been reported that when used acutely, the major target of rapamycin is mTOR present in complex 1 (TORC1), but chronic exposure to the drug results in additional inhibition of TORC2 (Lamming et al. 2012).

Based on studies in invertebrates using TOR and other mutants downstream in the pathway, as well as mice with deletion of the mTOR-regulated S6 kinase 1 (S6K1), it has been suggested that rapamycin acts as a dietary restriction mimetic (Selman et al. 2009). Furthermore, when treatment was initiated at 9 months of age, the ITP investigators reported that rapamycin decreased the age-related gain in body weight, again suggesting that the effects could be similar to those of DR. In mouse, rapamycin also has significant positive effects on healthspan, preventing the onset of many age-related diseases including Alzheimer’s, Parkinson’s, several types of natural and experimentally induced cancer, atherosclerosis, ventricular hypertrophy and others. At the physiopathological level, rapamycin has positive effects on age-related alterations in heart, liver, adrenal glands, endometrium, and tendon, as well as the age-dependent decline in spontaneous activity. Many of these effects are similar to those observed in mice under dietary restriction (DR). Rapamycin also has negative effects on the development of cataracts, as well as testicular atrophy and defects in glucose handling reminiscent of those observed in diabetes (Wilkinson et al. 2012).

In spite of these similarities at the physiological level, our previous data using gene arrays and metabolomics in male mice treated with rapamycin for 6 months showed that although 40% dietary restriction and 14 ppm rapamycin have a similar inhibitory effect on mTOR signaling, they have quite different effects on gene expression and metabolites, suggesting significant differences between dietary restriction and rapamycin at the molecular level (Fok et al. 2013, 2014a).

Several studies have shown that the life extension induced by rapamycin is sexually dimorphic, in that even though the effect is significant in mice of both sexes, lifespan extension is consistently larger in female than in male mice. For example the most recent study from the ITP showed that the lowest dose of rapamycin used (4.2 ppm) increased median longevity by 16% vs. 3% for females and males respectively. A similar dimorphism was observed at both the middle (14 ppm; 21% vs. 13%) and the highest dose (42 ppm; 26% vs. 23%) (Miller et al. 2014).

Taking in consideration these gender differences, in this work we compared male and female mice with respect to the expression of several genes involved in pathways that are known to play a role in longevity, and to be affected (based on our microarray data) by DR. The pathways we chose include: sirtuins, Foxo, circadian rhythm genes, degradation pathways (Ub-proteasome and autophagy), and the unfolding protein response. As in previous work (Fok et al. 2013; Fok et al. 2014a), we focused on the liver, but based on recent data from the ITP, we included female mice treated with three different doses of rapamycin (14 ppm, 22 ppm and 42 ppm) for 6 months starting at 5 months of age, and compared the results to female mice under DR (40%) for a similar length of time and at the same ages. Our data show that in females, effects of rapamycin on the expression of these genes closely resemble the effect of DR. In contrast, rapamycin at 14 ppm did not have any effect on the expression of these longevity-related genes in males. We did not examine males at the higher doses of rapamycin. These data may explain in part why females display a larger extension of lifespan than males in response to rapamycin treatment.

## Materials and methods

### Animals and feeding regiment

C57BL/6 mice were purchased from The Jackson Labs (Bar Harbor, ME) and placed on a commercial mouse chow, 7012 Teklad LM-450 (Harlan Laboratories, Madison, WI). At 5 months of age, the mice were separated into three dietary regimens: ad libitium (AL), 40% diet restriction (DR), and AL diet plus rapamycin in the food. The AL group was fed a commercial mouse chow, Purina Mills Test Diet Control #1810306 (Purina Mills, St. Louis, MO). The DR group was fed 40% less food than eaten by the AL mice. The male and female rapamycin group was fed the AL diet supplemented with 14 ppm of encapsulated rapamycin in the food as described Harrison et al. (2009). Mice were maintained on these dietary conditions until 11 months of age (6 months of treatment). For the female mice, there were two more groups of mice fed with 22 and 42 ppm of rapamycin, respectively. Mice were then euthanized (in the morning) by carbon dioxide and liver and subcutaneous adipose tissues were collected, snap frozen in liquid nitrogen, and stored at −80°C until used. All procedures followed the guidelines approved by the Institutional Animal Care and Use Committee at the University of Texas Health Science Center at San Antonio. (Kennedy et al. 2014).

### RNA processing

Total RNA from frozen liver (25 mg) or subcutaneous fat (100 mg) was extracted using RNeasy kit (Qiagen, Valencia, CA) following manufacturer’s protocols. RNA quality was assessed by Agilent Bioanalyzer (Agilent Technologies, Santa Clara, CA), and RNA quantity was determined using the Nanodrop (Thermo Scientific, Wilmington, DE). 1 μg of total RNA was then processed into cDNA by SuperScript® III First-Strand Synthesis System for RT-PCR (Life Technologies, Grand Island, NY) following manufacturer’s protocols. Primers were either obtained from RealTimePrimers.com (Elkins Park, PA), PrimerBank (http://pga.mgh.harvard.edu/primerbank, Wang and Seed, 2003) or designed using OligoPerfect™ Primer (Life Technologies, Grand Island, NY) and Primer-BLAST (NCBI). The primer pairs used are described in Additional file 1: Table S1. Quantitative real-time RT-PCR (qRT-PCR) was performed using SYBR Green PCR Master Mix (Life Technologies, Grand Island, NY) with detection by a StepOnePlus^™^ Real-Time PCR System (Applied Biosystem, Inc., Foster City, CA). Actin was used as a housekeeping control. The qRT-PCR results were analyzed using ΔΔ CT method. Data were expressed as relative fold change compared to ad libitum (AL).

The genes studied in this work were: Forkhead box protein O1(*Foxo1*), Sirtuin 1 (*Sirt1*), Sirtuin 3 (*Sirt3*), circadian locomotor output cycles kaput (*Clock*), cryptochrome 2(*cry2*), period homolog 1(*per1*), period homolog 2 (*per2*), aryl hydrocarbon receptor nuclear translocator-like (*Arntl*, or *Bmal*), Ubiquitin carboxyl-terminal hydrolase 2 (*Usp2*), 26S proteasome non-ATPase regulatory subunit 9 (*psmd9*), Microtubule-associated protein 1A/1B-light chain (*LC3*), *Beclin 1*, Lysosome-associated membrane protein 2 (*Lamp2*), heat shock 70 kDa protein 9 (*Hspa9*); protein disulfide isomerase family A, member 4 (*Pdia4*); 78 kDa glucose-regulated protein (*Grp78*) and calreticulin (*Calr*).

### Immunoblotting

Frozen liver tissue was homogenized with in ice-cold RIPA buffer (50 mM Tris–HCl, pH7.4, 1% Nonidet P-40, 1% Sodium deoxycholate, 0.15 M NaCl) supplemented with protease and phosphatase inhibitors (Roche, Indianapolis, IN) on ice. The supernatant was collected after centrifugation at 4°C, 12,000×*g* for 10 minutes. Proteins were separated by sodium dodecyl sulfate–polyacrylamide gel followed by transfer to PVDF membranes. Target proteins were detected with the following specific monoclonal or polyclonal antibodies: Actin (MP Biomedicals, Solon, OH); Bmal1 (Abcam, Cambridge, MA); Psmd9 (Sigma, St. Louis, MO). Grp78, Pdi, Sirt3 and LC3 from Cell signaling (Danvers, MA). Actin was quantified as a loading control. Images were analyzed by Imagelab software (Bio-rad, Hercules, CA). Data were expressed as means ± standard error of the mean (*SEM*).

### Statistical analysis

All data were expressed as mean ± standard error of the mean (SEM) of N = 6 to 8 samples per group, and were analyzed by analysis of variance followed by Fisher’s protected least-significant procedure. A p-value of less than 0.05 (two-tail) was considered significant.

## Results

**Figure 1:**
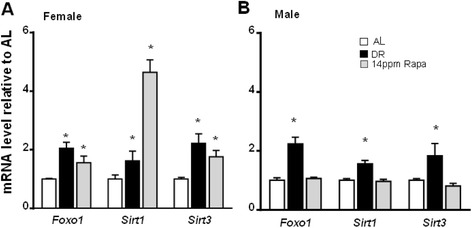
Rapamycin increases expression of Foxo and Sirtuin genes in females but not in males. The effect of dietary restriction (DR) and rapamycin (Rapa) on the expression of Foxo and Sirtuin genes was measured in liver samples from female **(A)** and male **(B)** mice. Open bars: AL; solid bars: DR; Gray bars: Rapa 14 ppm. The data were obtained from 6 male and 8 female mice per group and expressed as mean ± SEM. An asterisk denotes values that are significantly different (p ≤ 0.05) from AL mice.

In an effort to test the molecular basis for why the effect of rapamycin on longevity is stronger in females than males, we compared gene expression patterns in response to rapamycin (14 ppm, the initial dose shown to increase longevity in both male and female mice) in the liver of male and female mice, using as reference animals treated with DR (40%). Since a recent report by Miller et al., 2014 showed that the effect of rapamycin on longevity is dose dependent and the effect is still larger in females at all doses tested, changes in gene expression in females using the doses of 22 and 42 ppm were also evaluated. Our data showed that in female mice, rapamycin significantly upregulates the expression of *Foxo-1*, s*irtuin*-1 and 3 genes (Figure 1A), as observed for mice from either sex under DR. In fact, at 14 ppm rapamycin increased *Sirt1* gene expression more than DR. In contrast, male animals at that dose of rapamycin failed to increase expression of either of these genes (Figure 1B). We did not observe a dose-dependent effect in gene expression when female animals were fed with higher doses of rapamycin (22 or 42 ppm, Additional file 1: Figure S1A). In fact, the effect was more muted at higher doses of the drug.

**Figure 2:**
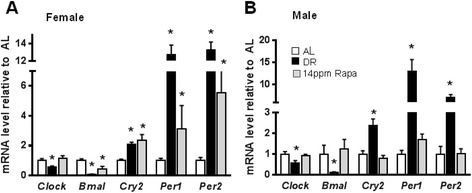
In female but not male mice, rapamycin and dietary restriction alter the expression of circadian rhythm genes similarly. The effect of dietary restriction (DR) and rapamycin (Rapa) on the expression of circadian rhythm genes was measured in liver samples from female **(A)** and male **(B)** mice. Open bars: AL; solid bars: DR; Gray bars: Rapa 14 ppm. The data were obtained from 6 male and 8 female mice per group and expressed as mean ± SEM. An asterisk denotes those values that are significantly different (p ≤ 0.05) from AL mice.

It is well established that circadian rhythm genes are altered by DR (Challet et al. 1998; Froy and Miskin 2007), which was also confirmed in this work, where the expression of genes involved in the circadian rhythm pathway (Figure 2), were significantly altered by DR in both males and females, including downregulation of the genes coding for *Clock* and *Bmal*, and overexpression of *Cry, Per1* and *Per2,* all of which are consistent with previous reports (Huang et al. 2011). With the exception of *clock* downregulation, qualitatively similar results were observed for expression of these genes in female mice treated with rapamycin (Figure 2A). Again however, rapamycin-fed male mice (14 ppm) did not show differences in the expression of any of these genes when compared to mice fed *ad libitum* (AL; Figure 2B), and no dose dependent-effect in any of these genes was observed in females (Additional file 1: Figure S1B).

**Figure 3:**
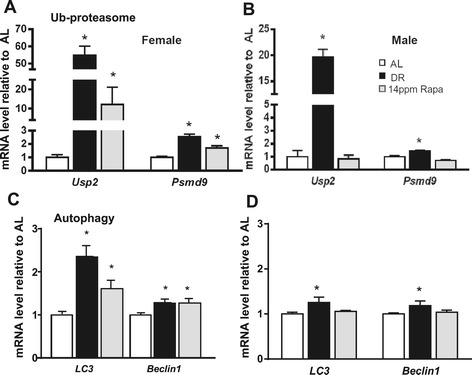
Rapamycin changed the expression of gene related to proteolytic pathways like dietary restriction only in female. The effect of dietary restriction (DR) and Rapamycin (Rapa), on the expression of genes related to ubiquitin-proteasome **(A and B)** and autophagy **(C and D)** pathways was measured in liver samples of male **(B and D)** and female **(A and C)** mice. The data were obtained from 6 male and 8 female mice per group and expressed as mean ± SEM. An asterisk denotes those values that are significantly different (p ≤ 0.05) from AL mice. Open bars: AL; solid bars: DR; Gray bars: Rapa 14 ppm.

Similar results were found when we measured the gene transcripts for key proteins involved in degradation processes i.e., ubiquitin-proteasome and autophagy pathways. For the ubiquitin-proteasome pathway, we measured mRNA levels for *Usp2* (ubiquitin specific peptidase 2), a cysteine protease that cleaves ubiquitin from the poly ubiquitin chains of proteins (Renatus et al., 2006; Stevenson et al. 2007) and *Psmd9*, a non-ATPase subunit of the 19S regulatory complex which aids in proteasome assembly and interacts with poly-ubiquitin chains of target proteins, influencing ubiquitination (Sangith et al., 2014). As shown in Figure 3A and B, DR in both genders significantly upregulates expression of both *Usp2* and *Psmd9* genes when compared to AL mice, an effect that is mimicked in females but not in males fed rapamycin. As before, the maximum effect on the expression of these genes was observed with rapamycin treatment at 14 ppm (Figure 3C).

For the autophagy pathway, we measured *Beclin-1 and LC3* genes, which code for proteins involved in the formation of double membrane autophagosomes, which are essential for macroautophagy. Again, expression of these genes was induced by DR in animals of both sexes, and by rapamycin only in females (Figure 3C and D). Rapamycin had no effect on the expression of these genes in males, and the effect in females was not dose-dependent (Additional file 1: Figure S1C and D). Since females showed an increase in the expression of these two macroautophagy genes, we also analyzed expression of the *Lamp2* gene, which codes for a lysosomal membrane protein involved in chaperone mediated autophagy. We found that both DR and rapamycin treatment had a similar effect in *Lamp2* expression in females, where both treatments increased its expression similarly (Additional file 1: Figure S2A).

**Figure 4:**
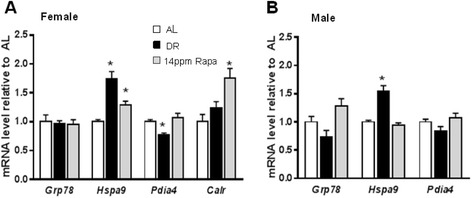
Rapamycin changes significantly the expression of genes involved in the ER stress pathway only in females. The effect of dietary restriction (DR) and rapamycin (Rapa) on the expression of genes involved in the ER stress pathway was measured in liver samples from female **(A)** and male **(B)** mice. The data were obtained from 6 male and 8 female mice per group and expressed as mean ± SEM. An asterisk denotes those values that are significantly different (p ≤ 0.05) from AL mice. Open bars: AL; solid bars: DR; Gray bars: Rapa 14 ppm.

Finally, we investigated the effect of rapamycin on the expression of selected genes involved in response to ER stress pathways, including the unfolded protein response (UPR) (Figure 4). For this pathway we measured the expression of *Grp78*, an ER chaperone (Li and Lee, 2006), *Hspa9,* a heat-shock cognate protein that plays an important role in the ER stress response, *Pdia4*, a folding enzyme localized in the ER lumen that also has chaperone activity to prevent protein aggregation (Thomas et al., 2010) and Calreticulin (*Calr*), a calcium binding chaperone protein in the ER lumen involved in folding of newly synthesized proteins (Saito et al., 1999). Figure 4A shows that in females neither DR nor Rapa (14 ppm) affected the levels of *Grp78* mRNA, but they both increased *Hspa9* and *Calr* gene expression. However, and contrary to DR, rapamycin had no effect on expression of the *Pdia4* gene.

In males (Figure 4B), DR produced a significant increase in the expression of *Hspa9*, and a non-significant decrease in the expression of *Grp78* and *Pdia4*. Rapamycin (14 ppm) did not have a significant effect on any of these mRNAs in male mice when compared to AL controls.

In females, expression of *Pdia4*, *Grp78* and *Calr* was maximal at a higher dose of rapamycin (22 ppm), and decreased at the highest dose (42 ppm). In contrast, expression of *Hspa9*, which is affected by 14 ppm, was not induced at the higher doses of rapamycin (22 and 42 ppm; Additional file 1: Figure S2B).

**Figure 5:**
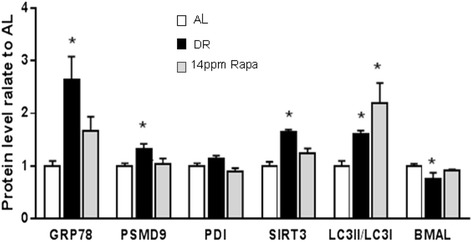
Effect of dietary restriction (DR) and rapamycin on protein levels in the liver. The effect of dietary restriction (DR) and rapamycin (Rapa) on protein levels was measured by Western blot analysis in livers obtained from 8 female mice per group and expressed as mean ± SEM. An asterisk denotes those values that are significantly different (p ≤ 0.05) from AL mice. Open bars: AL; Solid bars: DR; Gray bars: Rapa 14 ppm.

To identify whether these changes in gene expression are reflected in changes at the protein level, we used western blot analysis to assess the levels of representative proteins from each pathway (Figure 5). Our data show that in general there is a good correlation between changes in gene expression and protein levels under DR treatment. However, while we observed a similar trend for most of the proteins studied, the correlation between gene expression and protein levels under rapamycin treatment was weaker, and only one protein (LC3) showed statistically significant differences from control AL female mice. No dose effect of rapamycin was observed (Additional file 1: Figure S3).

**Figure 6:**
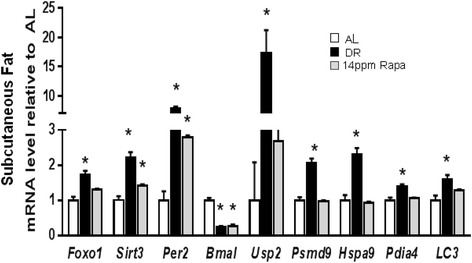
Rapamycin and DR exert similar effects on gene expression in subcutaneous fat from female mice. The effect of dietary restriction (DR) and rapamycin (Rapa) on gene expression was measured in subcutaneous fat from 6 female mice per group. The data was expressed as mean ± SEM, and an asterisk denotes those values that are significantly different (p ≤ 0.05) from AL mice. Open bars: AL; Solid bars: DR; Gray bars: Rapa 14 ppm.

To determine whether the changes in gene expression observed in liver represent a systemic effect of rapamycin in female mice, we used subcutaneous fat to measure the expression of those genes that showed the most significant changes in the liver. In Figure 6, we show that DR and rapamycin (14 ppm) affected the expression of most of the genes in a similar direction in subcutaneous fat, but the effect of rapamycin is significantly different from control group in only 3 genes of the 9 studied. Once again, we did not observe a rapamycin dose-effect in fat tissue (Additional file 1: Figure S4).

## Discussion

Several studies have indicated that the effect of rapamycin on longevity in mice is stronger in females than males. This includes studies in UM-HET-3 mice (Harrison et al. 2009; Miller et al. 2014), as well as our own studies in C57BL/6 mice, which showed that rapamycin started at 4 months (14 ppm) resulted in an increase in longevity of 11% for male and 16% for female C57BL/6 mice. Recently, a study by Zhang et al. (2014) showed that if rapamycin is started at 19 months of age, then only female C57BL/6 mice display an increase in lifespan (6%), while the treatment had no effect on longevity in male mice. More recently, Miller et al. showed that the extension of lifespan by rapamycin in UM-HET3 mice is dose dependent, and once again, the effect is larger in females than males at every dose tested (for 42 ppm, they found a significant increase of 26 and 23% respectively) (Miller et al. 2014). Thus it is clear from the literature that the effect of rapamycin on lifespan is sexually dimorphic.

In an effort to elucidate whether rapamycin and DR extend lifespan through similar mechanisms, in a previous study we used microarray and metabolomic analysis and showed that although treatment of male mice with either rapamycin (14 ppm) or DR (40%) for 6 months produces an overlapping set of changes in gene expression in the liver, rapamycin effects were generally weaker and it often showed no significant differences when compared to AL. Furthermore, there were no significant effects of rapamycin on a variety of liver metabolites measured, which was not the case for DR. These data were limited to only one gender, males, and since rapamycin has a larger longevity effect on females than in males, in this study we extended our observations to female mice and measured the effect of DR and rapamycin on the expression of several genes involved in pathways that are known to play a role in longevity. Generally, our data show that in females rapamycin had effects that are similar to those elicited by DR. For example, both interventions increased the expression of the transcription factor FoxO-1 as well sirtuins-1 and 3, which suggests that both DR and rapamycin may have a similar effect on cell metabolism, since both FoxO-1 and sirtuins play important roles in regulating glucose metabolism and cell death among other functions, and are known to play a key role in determining longevity (Puigserver et al. 2003; Boily et al. 2008; Hallows et al. 2011; Lopez-Otin et al. 2013). This possible effect on cell metabolism appears to be also corroborated by the similar effects of both rapamycin and DR on the expression of genes related to the circadian rhythm pathway, which is believed to play a role in healthspan (Wilkinson et al. 2012; Tevy et al. 2013), thus controlling the expression of several hormones that regulate metabolism and energy utilization. For example, clock genes are involved in regulating glucose metabolism in the liver (Rudic et al. 2004; Lamia et al. 2008; Zhang et al. 2010), and are essential for the maintenance of normal mitochondrial biogenesis and respiratory function in skeletal muscle (Andrews et al. 2010). Thus an up-regulation of circadian rhythm pathways would be consistent with increased longevity and improved healthspan, as observed in mice under DR and females fed rapamycin.

Our data also suggest that in females, both DR and rapamycin have a similar effect enhancing expression of genes involved in the ubiquitin-proteasome, autophagy, and UPR pathways. These pathways form part of the proteostasis network, which is generally believed to be one of the hallmarks of aging (Lopez-Otin et al. 2013; Burch et al. 2014; Kennedy et al. 2014; Morimoto and Cuervo 2014). Therefore, these data suggest that in females, both rapamycin and DR have a positive effect in regulating the protein quality control machinery, and also suggest that rapamycin may regulate stress resistance mechanisms, by inducing the expression of genes involved in the unfolding protein ER response, thus helping to take care of the proper folding of newly synthesized proteins.

Interestingly, genes that belong to the protein unfolding response were the only ones that showed a dose dependent effect with rapamycin, while DR had only a minor effect on genes belonging to this pathway: among the 4 genes we studied, only one was altered by DR (*Hspa9*). On the other hand, rapamycin altered all of them, and 3 of them were altered in a dose responsive fashion. This is relevant because it is known that, through specific binding to the immunophilin FKBP12, rapamycin inhibits the activity of mTOR and turns off protein synthesis (Thomson et al. 2009), which should result in a reduced stress at the ER level. However, when we estimated mTOR activity by measuring S6 phosphorylation, we observed a similar degree of mTOR inhibition between rapamycin and DR treatments (Fok et al., 2013) and thus a similar effect on both protein synthesis and protein quality control might be expected. As discussed, there are however significant differences between the effects of DR and rapamycin at the mRNA level (Figure 4). On the other hand, measurements at the protein level (Figure 5) indicate that in fact, at this level the effect of DR on proteostasis pathways is actually more robust than that observed after rapamycin treatment, with the exception of LC3 (I and II) protein, where DR and rapamycin have similar effects. Taken together, the data suggest that in female mice, both DR and rapamycin achieve the same improvement in protein quality control, but they appear to do so by different mechanisms: transcriptional in the case of rapamycin, and post-transcriptional in the case of DR.

Overall our data indicate that in contrast to the effect of DR, rapamycin treatment (14 ppm) did not alter gene expression in any of the chosen pathways in males. On the other hand, in females the same treatment (14 ppm rapamycin) did significantly change the expression of most of the genes studied in a fashion quite similar to DR. Also, our previous data indicate that even when fed rapamycin for 21 months, there were no change in microarray profiles in almost half of the male mice, suggesting that long-term feeding with rapamycin does not have a strong effect in males, or that the rapamycin’s effects at the transcriptional level may occur later that 21 months, since we nevertheless observed a difference in their mean lifespan (Fok et al. 2014b). However, it is possible that the data obtained from males fed 42 ppm would look similar to the data from females fed with rapamycin 14 ppm.

Our data also show that the effects on gene expression were not dependent on the dose of rapamycin, and this is in discordance with what has been observed for lifespan (Miller et al., 2014). Similar effects were found in subcutaneous fat tissue, where the expression of most of the genes was altered by both DR and rapamycin. However, in this tissue the effect of rapamycin was more variable and weaker than in liver, as shown by the fact that from the 9 genes studied, only 3 displayed significant changes, while 5 genes showed a tendency in the same direction as DR, but we did not reach statistical significance. The remaining gene, *Hspa9*, showed an increase with both DR and rapamycin in liver, but surprisingly, we found that its expression goes down with the higher concentration of rapamycin (but not DR) in fat. Another consideration is that higher rapamycin doses may regulate different pathways that are not activated by 14 ppm, and for that reason we do not observed a dose response effect. This information is not available, but it would be obtained by a global gene analysis like microarray studies.

Recently Miller et al., 2014, suggested that the gender difference on the effect of rapamycin might be due to the fact that females have higher blood levels of rapamycin than males. Although, we observed this difference in our animals too, with rapamycin blood level being significantly higher in females than males, we did not observe a significant difference in rapamycin levels in the liver, the tissue where most of our studies were done (Additional file 1: Figure S5A and B).

Therefore, based on these results and using a limited set of genes, we can speculate that in females, rapamycin has an effect that resembles the expectations for a DR mimetic more closely than in males, and this may explain in part why female mice respond better to rapamycin than male. Nevertheless, a deeper analysis must be done to understand the exact role played by each of these pathway(s) on the longevity effect mediated by rapamycin.

## Additional file

Additional file 1:
**Dose effect of rapamycin in gene expression (Figure S1, S2, S4), and protein levels (Figure S3) in the liver and subcutaneous fat of female mice.** All data was obtained from 8 female mice per group and expressed as mean ±SEM. An asterisk denotes those values that are significantly different (p ≤ 0.05) from AL mice. Open bar: AL; Solid bars: DR; Gray bars: Rapa 14 ppm, and striped bars represent different doses of rapamycin. **Figure S1.** Dose effect of rapamycin in the expression of *Foxo-1, Sirtuin*s genes (A), as well genes that belong to circadian rhythm (B) and proteolytic pathways (C, D) in the liver of female mice. **Figure S2.** Dose response in the expression of *Lamp 2* (A) and ER stress pathway (B) in the liver of female mice. **Figure S3.** Effect of different doses of rapamycin on protein levels in female liver. **Figure S4.** Effect of different doses of rapamycin on gene expression in subcutaneous fat from females. **Figure S5.** Intra-hepatic levels of rapamycin are similar between males and females. Rapamycin levels were measured in blood (A), and in liver tissue, expressed either as pg/mg of tissue (B), or relative to body weight (C). Open bars: Males; Solid bars: Females. The data was obtained from 8 female mice per group and expressed as mean ±SEM. Values were not statistically different (p ≤ 0.05) in any of the comparisons **Table S1.** Primers for qRT-PCR analysis. **Table S1.** list the primers used in the qRT-PCR analysis. The 5′ primer and the 3′ primer are listed.

## References

[CR1] Andrews JL, Zhang X, McCarthy JJ, McDearmon EL, Hornberger TA, Russell B, Campbell KS, Arbogast S, Reid MB, Walker JR, Hogenesch JB, Takahashi JS, Esser KA (2010). CLOCK and BMAL1 regulate MyoD and are necessary for maintenance of skeletal muscle phenotype and function. Proc Natl Acad Sci U S A.

[CR2] Boily G, Seifert EL, Bevilacqua L, He XH, Sabourin G, Estey C, Moffat C, Crawford S, Saliba S, Jardine K, Xuan J, Evans M, Harper ME, McBurney MW (2008). SirT1 regulates energy metabolism and response to caloric restriction in mice. PLoS One.

[CR3] Burch JB, Augustine AD, Frieden LA, Hadley E, Howcroft TK, Johnson R, Khalsa PS, Kohanski RA, Li XL, Macchiarini F, Niederehe G, Oh YS, Pawlyk AC, Rodriguez H, Rowland JH, Shen GL, Sierra F, Wise BC (2014). Advances in geroscience: impact on healthspan and chronic disease. J Gerontol A Biol Sci Med Sci.

[CR4] Challet E, Solberg LC, Turek FW (1998). Entrainment in calorie-restricted mice: conflicting zeitgebers and free-running conditions. Am J Physiol.

[CR5] Fok WC, Bokov A, Gelfond J, Yu Z, Zhang Y, Doderer M, Chen Y, Javors M, Wood WH, Becker KG, Richardson A, Perez VI (2014). Combined treatment of rapamycin and dietary restriction has a larger effect on the transcriptome and metabolome of liver. Aging Cell.

[CR6] Fok WC, Chen Y, Bokov A, Zhang Y, Salmon AB, Diaz V, Javors M, Wood WH, Zhang Y, Becker KG, Pérez VI, Richardson A (2014). Mice fed rapamycin have an increase in lifespan associated with major changes in the liver transcriptome. PLoS One.

[CR7] Fok WC, Zhang Y, Salmon AB, Bhattacharya A, Gunda R, Jones D, Ward W, Fisher K, Richardson A, Perez VI (2013). Short-term treatment with rapamycin and dietary restriction have overlapping and distinctive effects in young mice. J Gerontol A Biol Sci Med Sci.

[CR8] Froy O, Miskin R (2007). The interrelations among feeding, circadian rhythms and ageing. Prog Neurobiol.

[CR9] Hallows WC, Yu W, Smith BC, Devries MK, Ellinger JJ, Someya S, Shortreed MR, Prolla T, Markley JL, Smith LM, Zhao S, Guan KL, Denu JM (2011). Sirt3 promotes the urea cycle and fatty acid oxidation during dietary restriction. Mol Cell.

[CR10] Harrison DE, Strong R, Sharp ZD, Nelson JF, Astle CM, Flurkey K, Nadon NL, Wilkinson JE, Frenkel K, Carter CS, Pahor M, Javors MA, Fernandez E, Miller RA (2009). Rapamycin fed late in life extends lifespan in genetically heterogeneous mice. Nature.

[CR11] Huang W, Ramsey KM, Marcheva B, Bass J (2011). Circadian rhythms, sleep, and metabolism. J Clin Invest.

[CR12] Kennedy BK, Berger SL, Brunet A, Campisi J, Cuervo AM, Epel ES, Franceschi C, Lithgow GJ, Morimoto RI, Pessin JE, Rando TA, Richardson A, Schadt EE, Wyss-Coray T, Sierra F (2014). Geroscience: linking aging to chronic disease. Cell.

[CR13] Lamia KA, Storch KF, Weitz CJ (2008). Physiological significance of a peripheral tissue circadian clock. Proc Natl Acad Sci U S A.

[CR14] Lamming DW, Ye L, Katajisto P, Goncalves MD, Saitoh M, Stevens DM, Davis JG, Salmon AB, Richardson A, Ahima RS, Guertin DA, Sabatini DM, Baur JA (2012). Rapamycin-induced insulin resistance is mediated by mTORC2 loss and uncoupled from longevity. Science.

[CR15] Li J, Lee AS (2006). Stress induction of GRP78/BiP and its role in cancer. Curr Mol Med..

[CR16] Lopez-Otin C, Blasco MA, Partridge L, Serrano M, Kroemer G (2013). The hallmarks of aging. Cell.

[CR17] Miller RA, Harrison DE, Astle CM, Fernandez E, Flurkey K, Han M, Javors MA, Li X, Nadon NL, Nelson JF, Pletcher S, Salmon AB, Sharp ZD, Van Roekel S, Winkleman L, Strong R (2014). Rapamycin-mediated lifespan increase in mice is dose and sex dependent and metabolically distinct from dietary restriction. Aging Cell.

[CR18] Morimoto RI, Cuervo AM (2014). Proteostasis and the aging proteome in health and disease. J Gerontol A Biol Sci Med Sci.

[CR19] Puigserver P, Rhee J, Donovan J, Walkey CJ, Yoon JC, Oriente F, Kitamura Y, Altomonte J, Dong H, Accili D, Spiegelman BM (2003). Insulin-regulated hepatic gluconeogenesis through FOXO1-PGC-1alpha interaction. Nature.

[CR20] Renatus M, Parrado SG, D'Arcy A, Eidhoff U, Gerhartz B, Hassiepen U, Pierrat B, Riedl R, Vinzenz D, Worpenberg S, Kroemer M (2006). Structural basis of ubiquitin recognition by the deubiquitinating protease USP2. Structure..

[CR21] Rudic RD, McNamara P, Curtis AM, Boston RC, Panda S, Hogenesch JB, Fitzgerald GA (2004). BMAL1 and CLOCK, two essential components of the circadian clock, are involved in glucose homeostasis. PLoS Biol.

[CR22] Saito Y, Ihara Y, Leach MR, Cohen-Doyle MF, Williams DB (1999). Calreticulin functions in vitro as a molecular chaperone for both glycosylated and non-glycosylated proteins. EMBO J..

[CR23] Sangith N, Srinivasaraghavan K, Sahu I, Desai A, Medipally S, Somavarappu AK, Verma C, Venkatraman P (2014). Discovery of novel interacting partners of PSMD9, a proteasomal chaperone: Role of an Atypical and versatile PDZ-domain motif interaction and identification of putative functional modules. FEBS Open Bio..

[CR24] Selman C, Tullet JM, Wieser D, Irvine E, Lingard SJ, Choudhury AI, Claret M, Al-Qassab H, Carmignac D, Ramadani F, Woods A, Robinson IC, Schuster E, Batterham RL, Kozma SC, Thomas G, Carling D, Okkenhaug K, Thornton JM, Partridge L, Gems D, Withers DJ (2009). Ribosomal protein S6 kinase 1 signaling regulates mammalian life span. Science.

[CR25] Stevenson LF, Sparks A, Allende-Vega N, Xirodimas DP, Lane DP, Saville MK (2007). The deubiquitinating enzyme USP2a regulates the p53 pathway by targeting Mdm2. EMBO J..

[CR26] Tevy MF, Giebultowicz J, Pincus Z, Mazzoccoli G, Vinciguerra M (2013). Aging signaling pathways and circadian clock-dependent metabolic derangements. Trends Endocrinol Metab.

[CR27] Thomas M, George NI, Saini UT, Patterson TA, Hanig JP, Bowyer JF (2010). Endoplasmic reticulum stress responses differ in meninges and associated vasculature, striatum, and parietal cortex after a neurotoxic amphetamine exposure. Synapse..

[CR28] Thomson AW, Turnquist HR, Raimondi G (2009). Immunoregulatory functions of mTOR inhibition. Nat Rev Immunol.

[CR29] Wang X, Seed B (2003). A PCR primer bank for quantitative gene expression analysis. Nucleic Acids Res..

[CR30] Wilkinson JE, Burmeister L, Brooks SV, Chan CC, Friedline S, Harrison DE, Hejtmancik JF, Nadon N, Strong R, Wood LK, Woodward MA, Miller RA (2012) Rapamycin slows aging in mice. Aging Cell 11(4): 675–82 10.1111/j.1474-9726.2012.00832.x PMC3434687 22587563

[CR31] Zhang EE, Liu Y, Dentin R, Pongsawakul PY, Liu AC, Hirota T, Nusinow DA, Sun X, Landais S, Kodama Y, Brenner DA, Montminy M, Kay SA (2010). Cryptochrome mediates circadian regulation of cAMP signaling and hepatic gluconeogenesis. Nat Med.

[CR32] Zhang Y, Bokov A, Gelfond J, Soto V, Ikeno Y, Hubbard G, Diaz V, Sloane L, Maslin K, Treaster S, Rendon S, van Remmen H, Ward W, Javors M, Richardson A, Austad SN, Fischer K (2014). Rapamycin Extends Life and Health in C57BL/6 Mice. J Gerontol A Biol Sci Med Sci.

