# Supplementary material for: Gene expression in the liver of female, but not male mice treated with rapamycin resembles changes observed under dietary restriction
Source: Springerplus. 2015 Apr 11;4:174. doi: 10.1186/s40064-015-0909-7 (PMC4447730; doi:10.1186/s40064-015-0909-7)
Supplement: Additional file 1: — Dose effect of rapamycin in gene expression (Figure S1, S2, S4), and protein levels (Figure S3) in the liver and subcutaneous fat of female mice. All data was obtained from 8 female mice per group and expressed as mean ±SEM. An asterisk denotes those values that are significantly different (p ≤ 0.05) from AL mice. Open bar: AL; Solid bars: DR; Gray bars: Rapa 14 ppm, and striped bars represent different doses of rapamycin. Figure S1. Dose effect of rapamycin in the expression of Foxo-1, Sirtuins genes (A), as well genes that belong to circadian rhythm (B) and proteolytic pathways (C, D) in the liver of female mice. Figure S2. Dose response in the expression of Lamp 2 (A) and ER stress pathway (B) in the liver of female mice. Figure S3. Effect of different doses of rapamycin on protein levels in female liver. Figure S4. Effect of different doses of rapamycin on gene expression in subcutaneous fat from females. Figure S5. Intra-hepatic levels of rapamycin are similar between males and females. Rapamycin levels were measured in blood (A), and in liver tissue, expressed either as pg/mg of tissue (B), or relative to body weight (C). Open bars: Males; Solid bars: Females. The data was obtained from 8 female mice per group and expressed as mean ±SEM. Values were not statistically different (p ≤ 0.05) in any of the comparisons Table S1. Primers for qRT-PCR analysis. Table S1. list the primers used in the qRT-PCR analysis. The 5′ primer and the 3′ primer are listed. [file 40064_2015_909_MOESM1_ESM.pptx]

## Slide 1
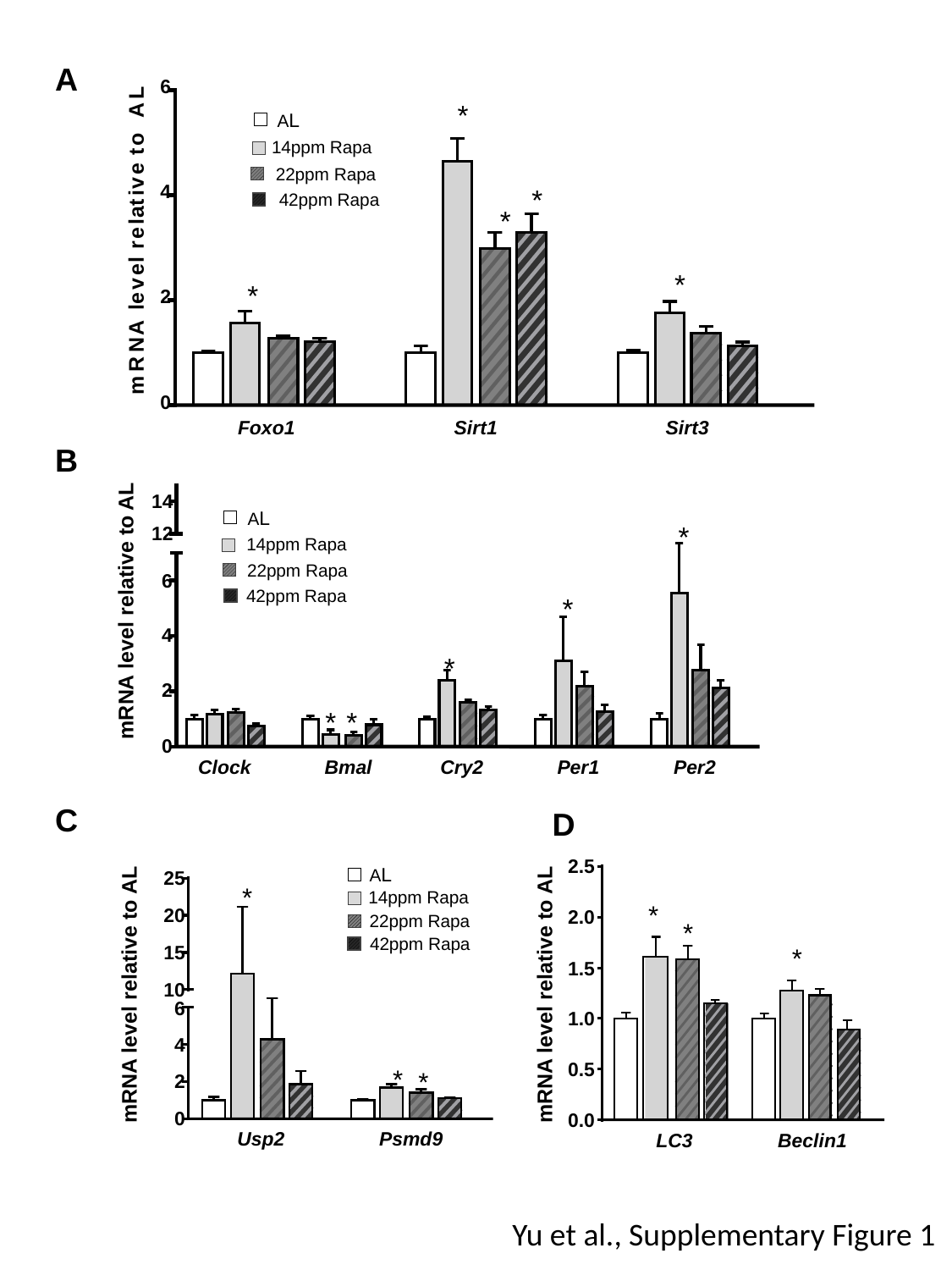

A
6
L
A
*
o
t
e
v
i
4
*
t
a
l
*
e
r
l
e
v
*
*
e
2
l
A
N
R
m
0
Foxo1
Sirt1
Sirt3
 AL
 14ppm Rapa
22ppm Rapa
42ppm Rapa
B
14
*
12
6
*
4
*
2
*
*
0
Clock
Bmal
Cry2
Per1
Per2
mRNA level relative to AL
 AL
 14ppm Rapa
22ppm Rapa
42ppm Rapa
C
D
25
 *
20
15
mRNA level relative to AL
10
6
4
*
*
2
0
Usp2
Psmd9
2.5
 *
2.0
*
*
1.5
1.0
0.5
0.0
LC3
Beclin1
mRNA level relative to AL
 AL
 14ppm Rapa
22ppm Rapa
42ppm Rapa
Yu et al., Supplementary Figure 1

## Slide 2
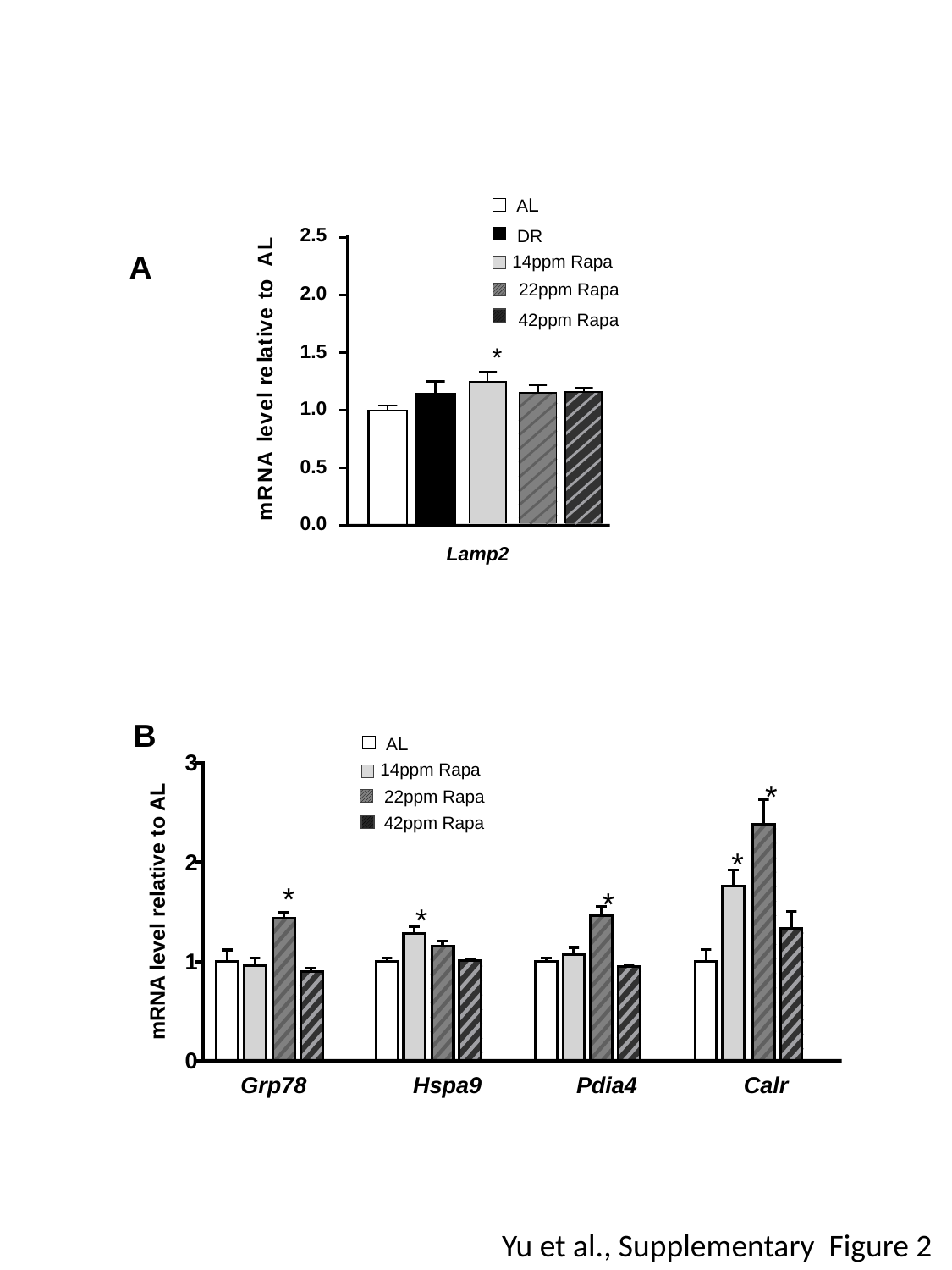

AL
 DR
 14ppm Rapa
 22ppm Rapa
 42ppm Rapa
L
2.5
A
o
t
2.0
e
v
i
t
a
l
1.5
*
e
r
l
e
v
1.0
e
l
A
N
0.5
R
m
0.0
Lamp2
A
B
 AL
 14ppm Rapa
22ppm Rapa
42ppm Rapa
3
*
*
2
*
*
mRNA level relative to AL
*
1
0
Grp78
Hspa9
Pdia4
Calr
Yu et al., Supplementary Figure 2

## Slide 3
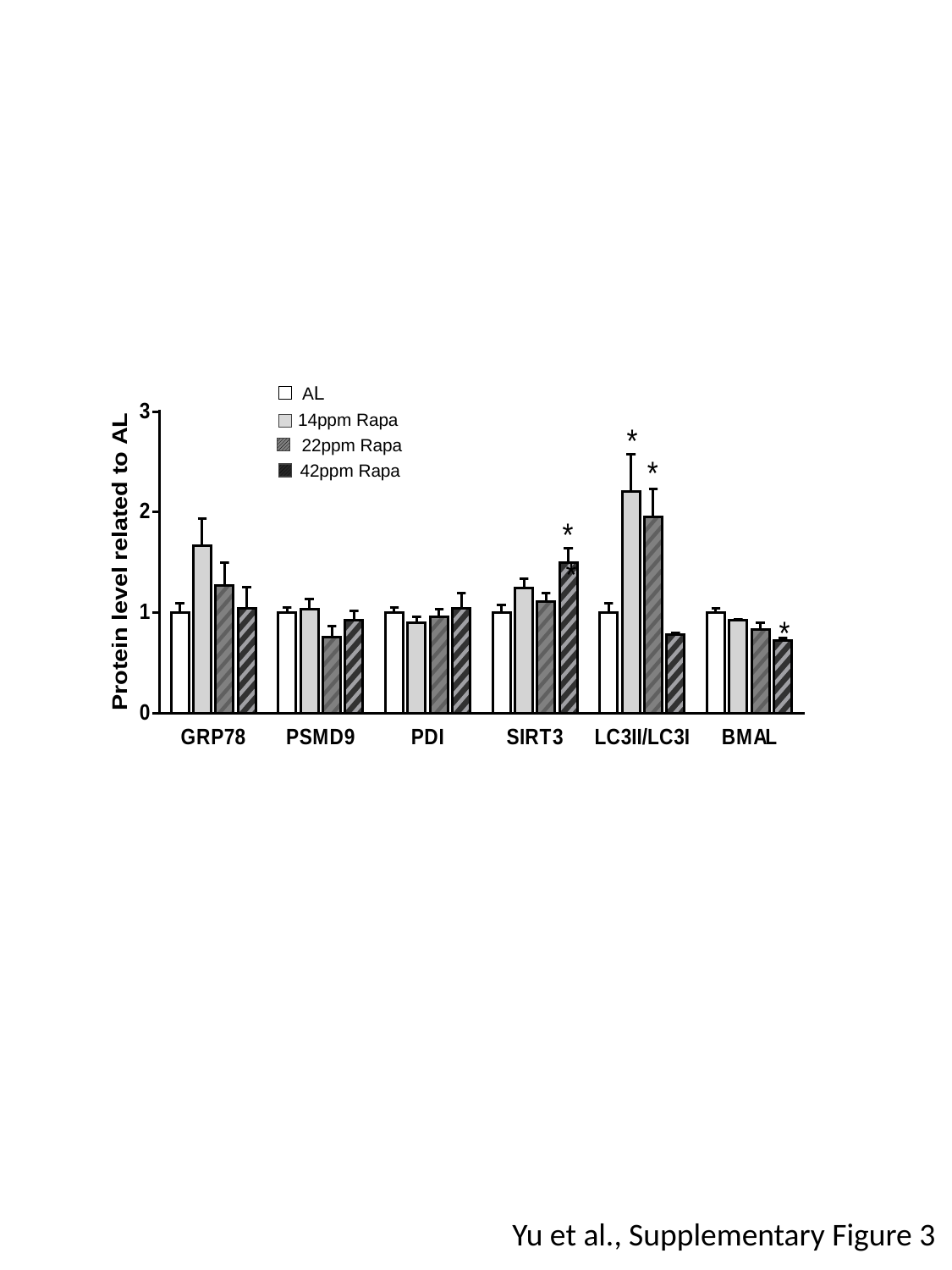

AL
 14ppm Rapa
22ppm Rapa
42ppm Rapa
Yu et al., Supplementary Figure 3

## Slide 4
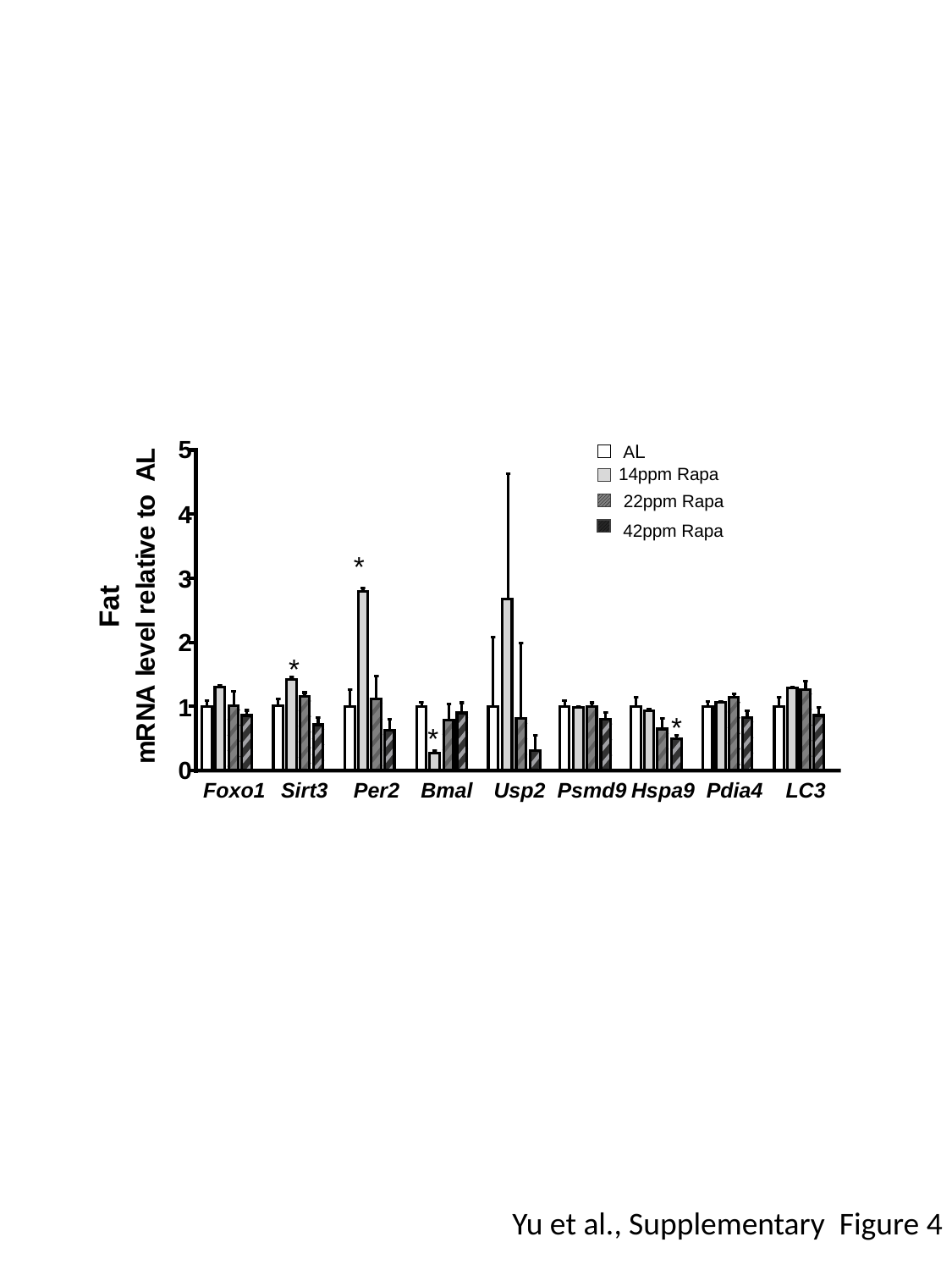

5
L
A
o
t
4
e
v
i
t
*
a
3
l
e
r
l
e
2
v
e
*
l
A
1
N
*
R
*
m
0
Foxo1
Sirt3
Per2
Bmal
Usp2
Psmd9
Hspa9
Pdia4
LC3
 AL
 14ppm Rapa
 22ppm Rapa
 42ppm Rapa
Fat
Yu et al., Supplementary Figure 4

## Slide 5
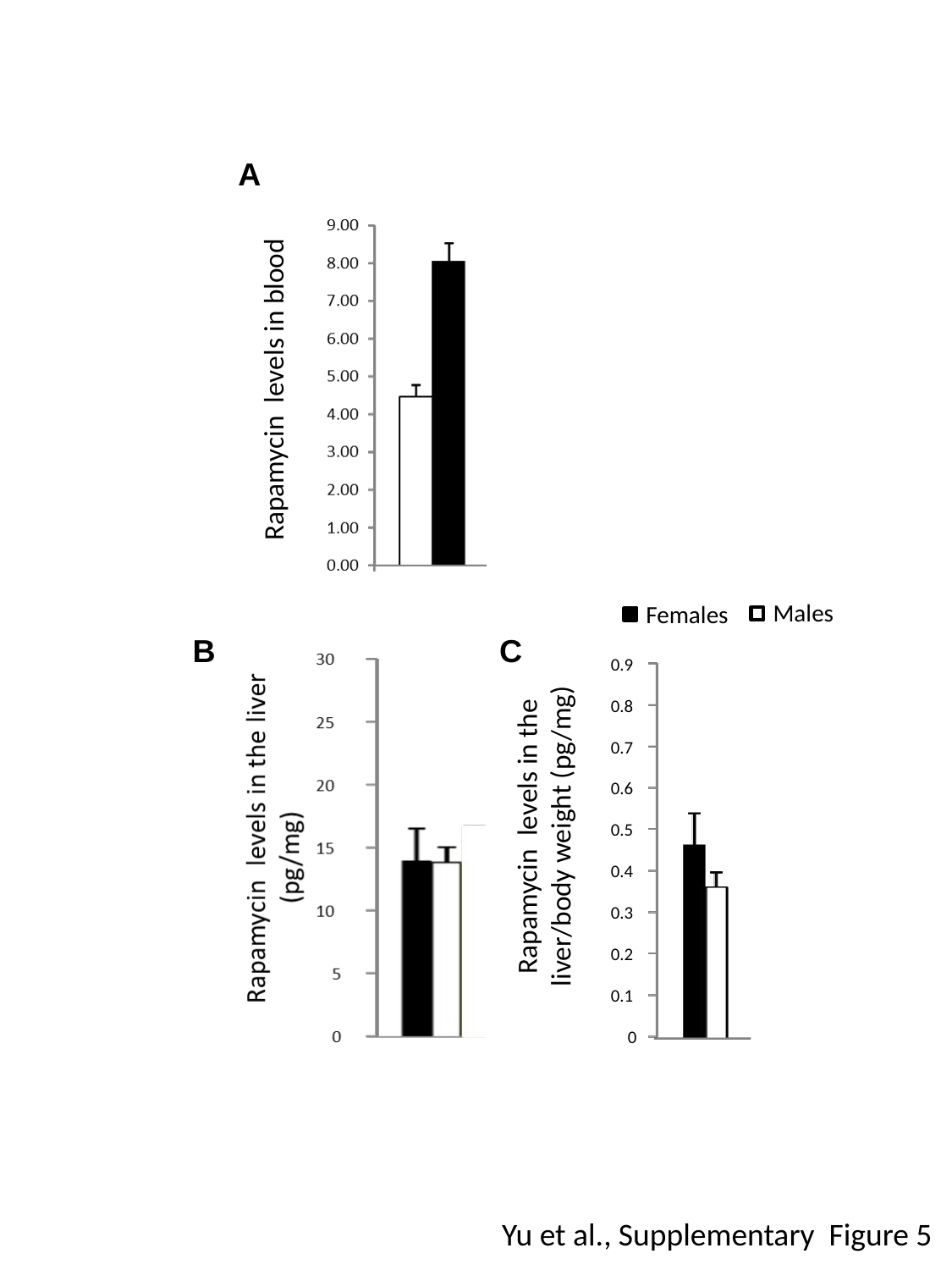

A
Rapamycin levels in blood
Males
Females
B
C
0.9
0.8
0.7
0.6
0.5
0.4
0.3
0.2
0.1
0
Rapamycin levels in the liver/body weight (pg/mg)
Yu et al., Supplementary Figure 5

## Slide 6
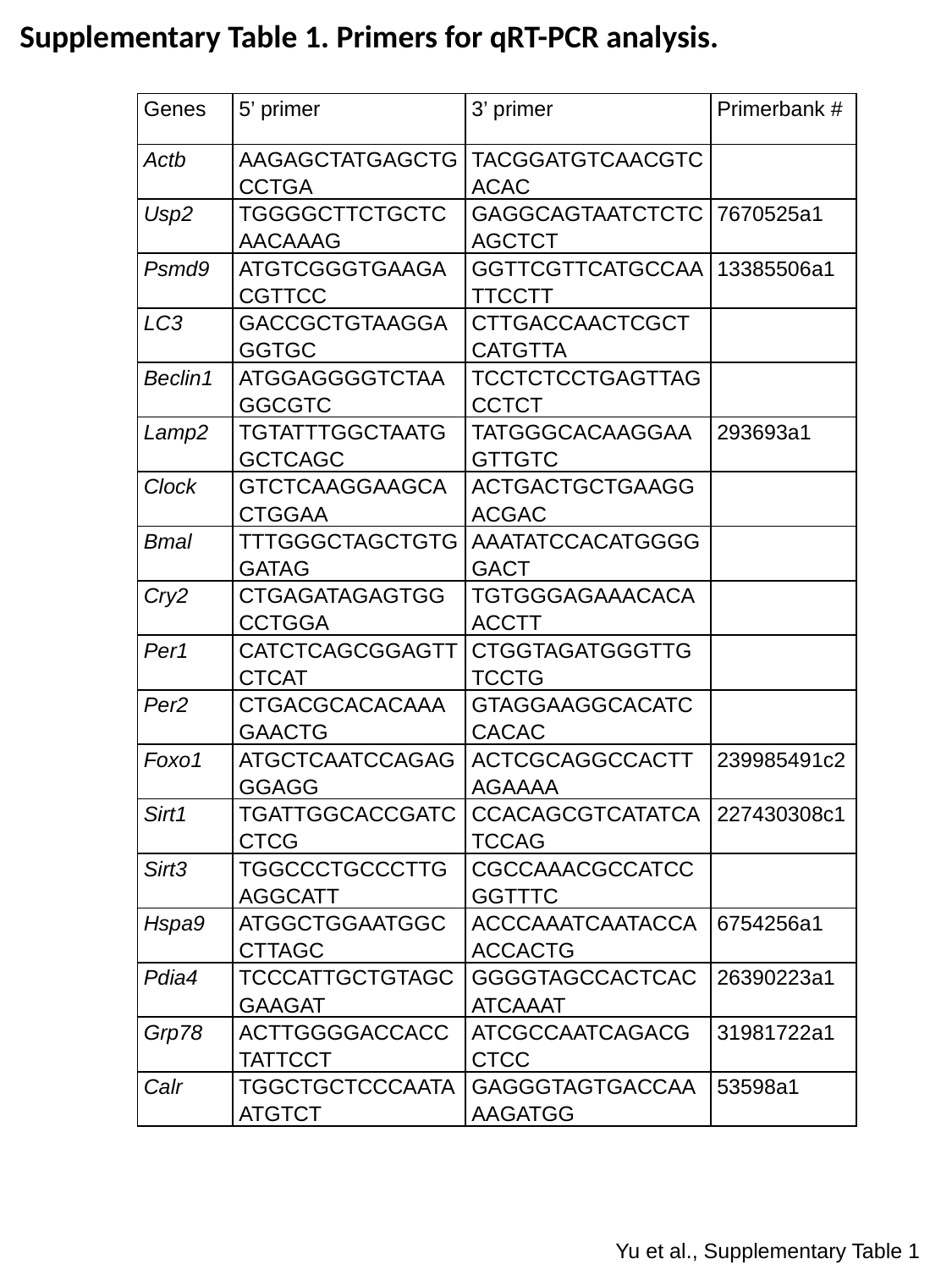

Supplementary Table 1. Primers for qRT-PCR analysis.
| Genes | 5’ primer | 3’ primer | Primerbank # |
| --- | --- | --- | --- |
| Actb | AAGAGCTATGAGCTGCCTGA | TACGGATGTCAACGTCACAC | |
| Usp2 | TGGGGCTTCTGCTCAACAAAG | GAGGCAGTAATCTCTCAGCTCT | 7670525a1 |
| Psmd9 | ATGTCGGGTGAAGACGTTCC | GGTTCGTTCATGCCAATTCCTT | 13385506a1 |
| LC3 | GACCGCTGTAAGGAGGTGC | CTTGACCAACTCGCTCATGTTA | |
| Beclin1 | ATGGAGGGGTCTAAGGCGTC | TCCTCTCCTGAGTTAGCCTCT | |
| Lamp2 | TGTATTTGGCTAATGGCTCAGC | TATGGGCACAAGGAAGTTGTC | 293693a1 |
| Clock | GTCTCAAGGAAGCACTGGAA | ACTGACTGCTGAAGGACGAC | |
| Bmal | TTTGGGCTAGCTGTGGATAG | AAATATCCACATGGGGGACT | |
| Cry2 | CTGAGATAGAGTGGCCTGGA | TGTGGGAGAAACACAACCTT | |
| Per1 | CATCTCAGCGGAGTTCTCAT | CTGGTAGATGGGTTGTCCTG | |
| Per2 | CTGACGCACACAAAGAACTG | GTAGGAAGGCACATCCACAC | |
| Foxo1 | ATGCTCAATCCAGAGGGAGG | ACTCGCAGGCCACTTAGAAAA | 239985491c2 |
| Sirt1 | TGATTGGCACCGATCCTCG | CCACAGCGTCATATCATCCAG | 227430308c1 |
| Sirt3 | TGGCCCTGCCCTTGAGGCATT | CGCCAAACGCCATCCGGTTTC | |
| Hspa9 | ATGGCTGGAATGGCCTTAGC | ACCCAAATCAATACCAACCACTG | 6754256a1 |
| Pdia4 | TCCCATTGCTGTAGCGAAGAT | GGGGTAGCCACTCACATCAAAT | 26390223a1 |
| Grp78 | ACTTGGGGACCACCTATTCCT | ATCGCCAATCAGACGCTCC | 31981722a1 |
| Calr | TGGCTGCTCCCAATAATGTCT | GAGGGTAGTGACCAAAAGATGG | 53598a1 |
Yu et al., Supplementary Table 1
